# CO_2_ and N_2_O Emissions from Spring Maize Soil under Alternate Irrigation between Saline Water and Groundwater in Hetao Irrigation District of Inner Mongolia, China

**DOI:** 10.3390/ijerph16152669

**Published:** 2019-07-25

**Authors:** Yu Wang, Peiling Yang, Shumei Ren, Xin He, Chenchen Wei, Shuaijie Wang, Yao Xu, Ziang Xu, Yanxia Zhang, Hassan Ismail

**Affiliations:** 1 College of Water Resources and Civil Engineering, China Agricultural University, Beijing 100083, China; 2 Department of Civil and Environmental Engineering, Penn State University, University Park, PA 16801, USA; 3 Henan Water Conservancy Investment Group Co., Ltd., Zhengzhou 450000, China; 4 Gongnongqu Electric Pumping Station, Xigu District, Lanzhou 730060, China

**Keywords:** irrigation water salinity, alternate irrigation regime, greenhouse gas emission, soil properties, global warming potential

## Abstract

Alternative irrigation between saline water and groundwater can alleviate shortages of available agricultural water while effectively slowing the adverse effects of saline water on the soil-crop system when compared with continuous irrigation with saline water and blending irrigation between saline water and groundwater. In 2018, we tested the effect on soil CO_2_ and N_2_O emissions by two types of irrigation regimes (alternating groundwater and saline water (GW-SW), and alternating groundwater, followed by two cycles of saline water (GW-SW-SW)) between groundwater and three levels of salinity of irrigation water (mineralization of 2 g/L, 3.5 g/L, and 5 g/L), analyzed the correlation between gas emissions and soil properties, calculated comprehensive global warming potential (GWP), and investigated the maize yield. The results show that, with the same alternate irrigation regime, cumulative CO_2_ emissions decreased with increasing irrigation water salinity, and cumulative N_2_O emissions increased. Cumulative CO_2_ emissions were higher in the GW-SW regime for the same irrigation water salinity, and cumulative N_2_O emissions were higher in the GW-SW-SW regime. The GW-SW-SW regime had less comprehensive GWP and maize yield as compared to the GW-SW regime. The 2 g/L salinity in both regimes showed larger comprehensive GWP and maize yield. The 3.5 g/L salinity under the GW-SW regime will be the best choice while considering that the smaller comprehensive GWP and the larger maize yield are appropriate for agricultural implication. Fertilizer type and irrigation amount can be taken into consideration in future research direction.

## 1. Introduction

The world’s major greenhouse gas (GHG) emissions reached a new high in 2017, with concentrations of 405.5 ± 0.1 ppm CO_2_ [1], 1859 ± 2 ppb CH_4_ [2], and of 329.9 ± 0.1 ppb N_2_O [3]. China is the leading GHG emitter, and CH_4_ and N_2_O emissions from the agriculture industry are 37.59% and 74.71% of the total GHG emissions in 2008, respectively [4,5], with the CO_2_ emissions reaching as much as 54.16 Metric ton (Mt) in 2010 [6].

Hetao Irrigation District, which is located in the midstream of the Yellow River, is one of the three largest irrigation areas in China covering an area of 11,600 km^2^ with 500 km^2^ of saline-alkaline soil. As an arid northwest plateau of China, the area is characterized by low rainfall and high evaporation. Without irrigation being drawn from the Yellow River, the agricultural industry in Hetao Irrigation District would be non-existent. With increases in economic development, the demand for industrial water and domestic water has increased, leading to strains on available water for agricultural use. Using unconventional water sources (e.g., saline water, wastewater, etc.) for agricultural use has been explored [7,8,9]. Combining unconventional water sources with drip irrigation and other efficient water-saving technologies can not only reduce the demand for freshwater in agricultural production, but can also effectively alleviate the water shortage in Hetao Irrigation District, which is rich in diverse saline water. As the largest design irrigation area in China, it is necessary to focus on GHG emissions from agricultural soil in Hetao Irrigation District.

In literature, some researchers focused on the interaction of GHG emissions and salt from soil or water [10,11,12]. Yang, et al. [13] studied the saline-alkaline soil and found that, when compared with low saline-alkaline soil, high saline-alkaline soil significantly increased Global Warming Potential (GWP) by raising N_2_O emission and reducing CH_4_ uptake. Tang, et al. [14] focused on saline–alkaline paddy fields and suggested that intermittent irrigation would be a better regime to weaken the combined GWP of CH_4_ and N_2_O. Zhang, et al. [15] irrigated a cotton field with saline water and found that saline water irrigation will stimulate N_2_O emission. Morales-Garcia, Stewart, Seguin, and Madramootoo [8] studied supplemental saline drip irrigation that was applied at different growth stages of two bell pepper cultivars in non-saline soil and suggest that this can be used while avoiding yield reductions. The previous studies did not focus on the GHG emission under the coupling of saline water and alkaline soil, but laid the foundation for the present study by showing the relationship between GHG emissions and salt from soil or water.

Continuous irrigation, blending irrigation, and alternate irrigation are three major irrigation regimes. Some researchers found continuous irrigation with saline water, whose irrigation water is saline water always, affected the physical and chemical properties of soil, especially accumulation effect of salt over multiple years, and considerably depressed both vegetative growth and yield [9,16]. Blending irrigation with saline water and groundwater, whose irrigation water is suitable saline water for irrigation obtained from blending high salinity water and groundwater, can slow soil salinization. However, premixing these sources of water is labor intensive. The irrigation waters of alternate irrigation are saline water and groundwater, alternating irrigate under certain regime. Li, et al. [17] found that alternate irrigation was more efficient than blending irrigation in alleviating the adverse effects of saline water on the soil-crop system. Accordingly, there is a possibility of applying alternate irrigation more broadly.

Maize is the main food crop in Hetao Irrigation District. In 2012, the area planted with maize in Hetao Irrigation District was 340,000 hm^2^, accounting for 14.5% of the maize planting area in Inner Mongolia, with a total output of 2.96 million tons and an average yield of 8706 t·hm^−2^, which accounted for 21% of the total maize production in Inner Mongolia [18]. To the best of the authors’ knowledge, a study on the combined effects of different alternate irrigation regimes between saline water and groundwater on spring maize soil has not been previously conducted to study GHG emissions.

This work presents results from a study that furthers the short-term 2017 field study at Hetao Irrigation District on the effect of alternate irrigation between saline water and groundwater [19]. The 2017 study that found both CO_2_ and N_2_O emissions were the highest in the plots that were treated with low salinity water and lowest in those that were treated with high salinity water. The researchers also found that using two saline water cycles resulted in less CO_2_ and N_2_O emissions than only one cycle. Regarding maize yield, the 2017 study found 2 g/L water salinity under one saline cycle had the higher maize yield. To continue the experiment from 2017, the present work was conducted while considering multi-year effects of the irrigation strategies. The GHG emissions from spring maize soil under two types of alternate irrigation regimes between groundwater and three levels of saline irrigation water were measured. The analysis included finding correlations to soil properties and estimation of comprehensive GWP and maize yield. The objective of this study is to identify the best choice for agricultural production based on comprehensive greenhouse warming potential and maize yield. This study provides a scientific basis for promoting the use of unconventional water resources and reducing the agricultural greenhouse gas emissions.

## 2. Materials and Methods

### 2.1. Study Site

**Figure 1:**
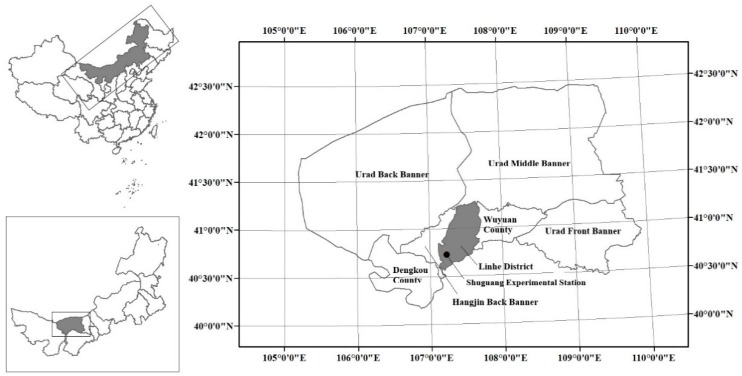
The location of Shuguang Experimental Station in China.

The field experiments were carried out from April to September in 2018 in Shuguang Experimental Station, Linhe District, Bayannaoer City, Inner Mongolia Autonomous Region (107°23’ E, 40°72’ N). Figure 1 shows the location of Shuguang Experimental Station in China. This area is located in the hinterland of Hetao Irrigation District. It is in the middle temperate and arid climate zones. Low precipitation, low wind, and dry with annual precipitation of only 141.2 mm characterize the site. Temperatures vary widely from day to night, with an average temperature of 6.8 °C. The frost-free period lasts for approximately 140 days. There are two types of soil within 1 m of the ground surface: silty loam with a bulk density of 1.49 g/cm^3^ and field capacity of 19.8% from 0–0.6 m below the ground surface, and silty clay loam with a bulk density of 1.44 g/cm^3^ and field capacity of 28.4% from 0.6–1 m depth.

### 2.2. Experimental Design

The experiment was arranged in a 3 × 2 full factorial experimental design, which corresponded to three levels of irrigation water salinity (mineralization of 2 g/L, 3.5 g/L, and 5 g/L, represented by S1, S2, and S3, respectively) and two types of alternate irrigation regimes: alternating groundwater and saline water (GW-SW) and alternating groundwater, followed by two cycles of saline water (GW-SW-SW), represented by L1 and L2, respectively. For the L1 cases, on watering days 1, 3, 5, etc., groundwater was used for watering, and on watering days 2, 4, 6, etc., saline water was used. For the L2 cases, groundwater was used for watering on days 1, 4, 7, etc., and saline water was used on days 2, 3, 5, 6, etc. Therefore, there were six treatments in total for all three irrigation water salinities, combined with each of the irrigation regimes, each with three replications. All 18 plots were randomly distributed, with a 1 m separation distance. The saline water that was used in the test was achieved by adding 1:2 molar ratio of potassium chloride (KCl) and sodium chloride (NaCl) to the local groundwater (mineralization of 1.157 g/L). During the experimental period, all of the treatments were treated with the same amount of nitrogen fertilizer and field management, which were based on the local cropping regime and farmer fertilization practices.

**Figure 2:**
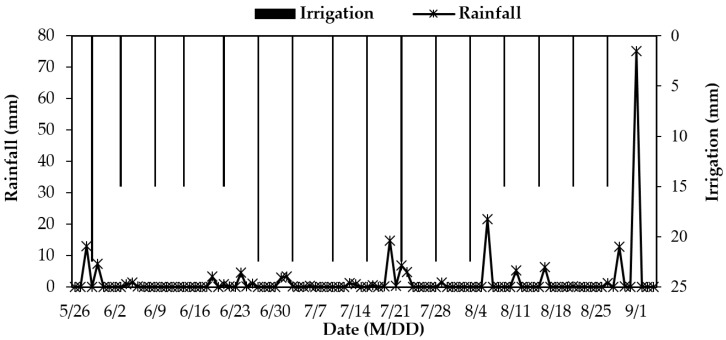
Irrigation and rainfall during the spring maize growth period in 2018.

The maize type that locals grow, “Simon 3358”, was used as the experimental object. Drip irrigation under a plastic film membrane was used in the study. The distance between two adjacent membranes was 0.7 m. The distance between two adjacent plants was 0.3 m. The distance between two rows of plants was 0.4 m. The drip irrigation lines were laid between rows with a wall thickness of 0.004 m, and the working flow rate of the drippers on the drip line was 2 l/h at a distance of 0.3 m. The base fertilizer with diammonium phosphate (375 kg·hm^−2^) and urea (75 kg·hm^−2^) was applied on 28 April 2018, which was six days before sowing. Topdressing was carried out in a ratio of 2:2:1 in the jointing stage (21 June 2018), large trumpet period (10 July 2018), and heading stage (9 August 2018), and the total amount of additional nitrogen fertilizer was 217.7 kg·hm^−2^. Proportional pumps were used to fertilize while watering. The total irrigation of the maize was 0.3 m applied 16 times through the testing period. The first watering was carried out at the seedling stage, and all of the plots were irrigated with groundwater. Figure 2 shows the irrigation schedule during the spring maize growth period.

### 2.3. Sample Collection and Measurement

**Figure 3:**
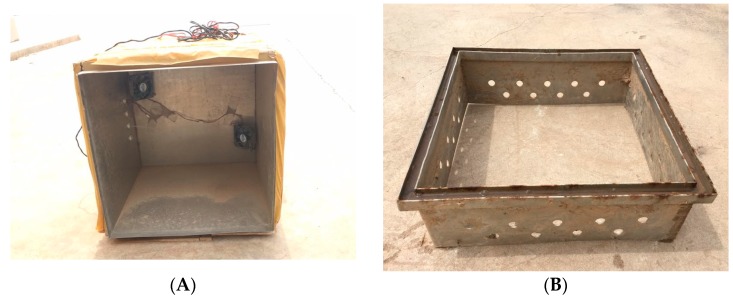

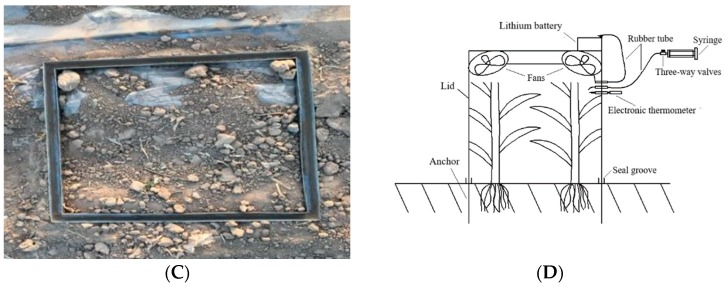
Structure of the static closed chamber showing (**A**) the lid, (**B**) the anchor, (**C**) the top view of the anchor embedded in the plot, and (**D**) the side view of the static closed chamber working.

The static closed chamber technique was used to collect the gas samples [20]. Figure 3 shows the structure of the static closed chamber that was used in the present study. The static closed chamber consisted of a lid (0.5 m × 0.5 m × 0.5 m) and an anchor (0.5 m × 0.5 m × 0.15 m). The lid was a stainless steel cube with an open bottom (Figure 3A). The lid was externally wrapped with 0.03 m thick plastic foam and tape. Two small fans were installed in the body to mix the gas in the chamber. The anchor was also stainless steel with an open top and bottom (Figure 3B). The anchor was embedded in each plot before the crop was sowed (Figure 3C). Water was poured into the seal groove of the anchor before collecting the gas, and the lid was covered to ensure airtightness.

During the growth period, gas samples were collected every seven days, as well as the first, third, fifth, and seventh days after fertigation. A polystyrene syringe with a three-way valve was used to extract 0.05 L of gas from the chamber at 0, 10, 20, and 30 min. after covering the lid. The gas samples were collected between at 8:30–11:00 am, since the soil temperature was close to the average daily temperature at that time. The temperature of the gas was measured at the same time while using an electronic thermometer. All of the gas samples were immediately sent to the laboratory and concentrations were measured by gas chromatography (Agilent Technologies, Agilent 7890A, Santa Clara, CA, USA).

Emissions are significantly affected by soil properties in the first few centimeters below the soil surface due to gas production and diffusion [21]. Soil samples from the ground surface to 0.3 m depth were taken via a soil drill four times at the jointing stage (30 June 2018), large trumpet period (12 July 2018), heading stage (13 August 2018), and maturing period (15 September 2018) with three repetitions per treatment. Soil moisture was calculated by the drying method. A portable multi-parameter meter measured the electrical conductivity (EC) and pH (Mettler Toledo, SG23-FK-CN, Columbus, OH, USA), while NH_4_^+^-N concentration and NO_3_^−^-N concentration were determined by a continuous flow analyzer (Alliance FUTURA, AMS, Frépillon, France).

### 2.4. Calculation Method of Gas Emission

The daily gas emission flux was calculated by the following Equation (1) [22]:

(1) $$F=\rho \times \left(\frac{V}{A}\right)\times \left(\frac{\Delta c}{\Delta t}\right)\times \left(\frac{273}{T}\right)$$

F refers to the daily gas emission flux (M·L^−2^·T^−1^); ρ is the gas density in the standard state (M·L^−3^); V and A are the volume and the area of the lid, respectively (L^3^ and L^2^, respectively); ∆c⁄∆t refers to the linear change in gas concentration with time (L·L^−1^·T^−1^); and, T is the Kelvin temperature of the gas (K).

The daily gas emission flux during the maize growth period (from 27 May to 15 September 2018) is necessary for calculating cumulative gas emission. Daily gas emissions on non-measured days were obtained through linear interpolation since daily gas emission flux was measured frequently (every seven days) [23]. The following Equation was used to interpolate the non-measured daily flux data from the measured days:

(2) $$F_{L}=F_{1}-\left(\frac{F_{1}-F_{2}}{t_{2}-t_{1}}\right)\left(t_{L}-t_{1}\right)$$

F_L_ is the non-measured daily gas emission flux using linear interpolation (M·L^−2^·T^−1^); F_1_ is the daily gas emission flux measured on the latest date before the non-measured day; F_2_ is the daily gas emission flux measured on the earliest date after the non-measured day; and, t_1,_ t_2_, and t_1_ are the days F_1,_ F_2_, and F_L_ were measured, respectively. The daily gas emission fluxes in 2017 were obtained from a 2017 experiment [19].

### 2.5. Determination of the GWP

Global warming potential (GWP) can be used to evaluate the relative ability of various greenhouse gases to influence climate change. The GWP of N_2_O flux is 265 based on the units of CO_2_ equivalents during a 100-year time horizon [24]. The comprehensive GWP of the experiment can be calculated by the Equation below:

(3) $$\mathrm{GWP}={\mathrm{GWP}}_{{\mathrm{CO}}_{2}}+{\mathrm{GWP}}_{N_{2}O}=M\left({\mathrm{CO}}_{2}\right)\times 1+M\left(N_{2}O\right)\times 265$$

${\mathrm{GWP}}_{{\mathrm{CO}}_{2}}$ [kg(CO_2_-C)·hm^−2^] and ${\mathrm{GWP}}_{N_{2}O}$ [kg(CO_2_-C)·hm^−2^] are the GWP of CO_2_ and N_2_O, respectively. M(CO_2_) and M(N_2_O) are the accumulative CO_2_ emission and cumulative N_2_O emission during the growth period, respectively.

Among intensive cropping systems, CH_4_ typically shows a much smaller proportion and variance in GWP [25,26], so it was not measured or included in comprehensive GWP.

### 2.6. Statistical Analysis

Pearson correlation was performed to test the correlations between the mean gas emissions and the mean soil properties of the four growth periods. Statistical analyses were performed while using SPSS statistical software (version 11.5, SPSS Inc., Chicago, IL, USA, 2003). Repeated measures analysis of variance (ANOVA) was performed to test the difference in cumulative gas emission, comprehensive GWP, and maize yield between treatments at a significance level of 0.05. Irrigation water salinity and alternate irrigation regime were the independent variables. A Duncan multiple range test was performed to determine whether these two independent variables had significant effects (*p* < 0.05) on treatments.

## 3. Results

### 3.1. Soil Properties

**Figure 4:**
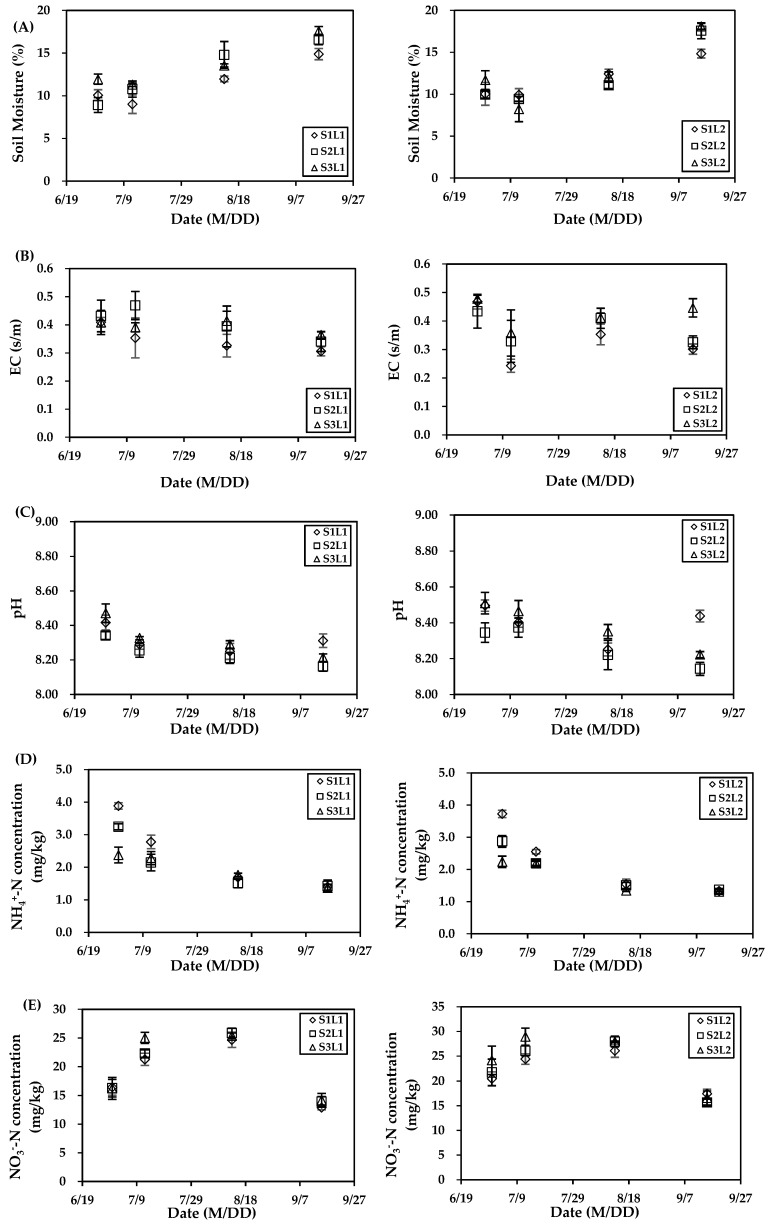
Variation of (**A**) soil moisture, (**B**) electrical conductivity—(EC), (**C**) pH, (**D**) NH_4_^+^-N concentration, and (**E**) NO_3_^−^-N concentration for different irrigation water salinity (S1, S2, and S3 for 2 g/L, 3.5 g/L, and 5 g/L, respectively) and alternate irrigation regimes (L1 and L2 for one saline water cycle and two saline water cycles, respectively). Symbols represent the mean value of the three repeated tests, and error bars represent standard deviation.

Figure 4 shows soil properties including moisture, EC, pH, NH_4_^+^-N concentration, and NO_3_^−^-N concentration for all the treatments in the four main growth periods.

The peak soil moisture for all treatments appeared on 15 September 2018 due to a large rainfall event after all irrigation was completed (Figure 4A). In the L1 plots, the average soil moistures were 11.49%, 12.77%, and 13.59% in S1, S2, and S3, respectively. In the L2 plots, the average soil moistures were 11.81%, 12.00%, and 12.53% in S1, S2, and S3, respectively. When compared to the L2 plots, the average soil moistures in the L1 plots were greater in general. The differences of S1, S2, and S3 were −2.76%, 6.42%, and 8.44%, respectively.

Soil salinity depends on the combination of the initial soil salinity and the irrigation water salinity. When compared to the early growing period, the EC in most treatments on September 15 was lower (Figure 4B), which may be due to the downward transport of salt during water transport. The highest average soil salinity in the L1 and L2 plots appeared in S2 (0.41 s/m) and S3 (0.42 s/m), respectively.

The pH reduced during the growth period, and all of the values were greater than 8.00, representing weak alkalinity (Figure 4C). The maximum mean pH appeared in S1L2 (8.40) and it was 2% larger than the minimum mean pH of 8.24 for S2L1.

NH_4_^+^-N and NO_3_^−^-N concentrations were lower at the end of the growing period (Figure 4D,E). When compared to 30 June 2018, the NH_4_^+^-N concentrations on 15 September 2018 reduced by 42.26–63.94% in the L1 plots and by 41.40–64.26% in the L2 plots; NO_3_^−^-N concentrations reduced by 12.77–21.50% in the L1 plots and by 14.77–31.41% in the L2 plots.

### 3.2. Daily Gas Flux

**Figure 5:**
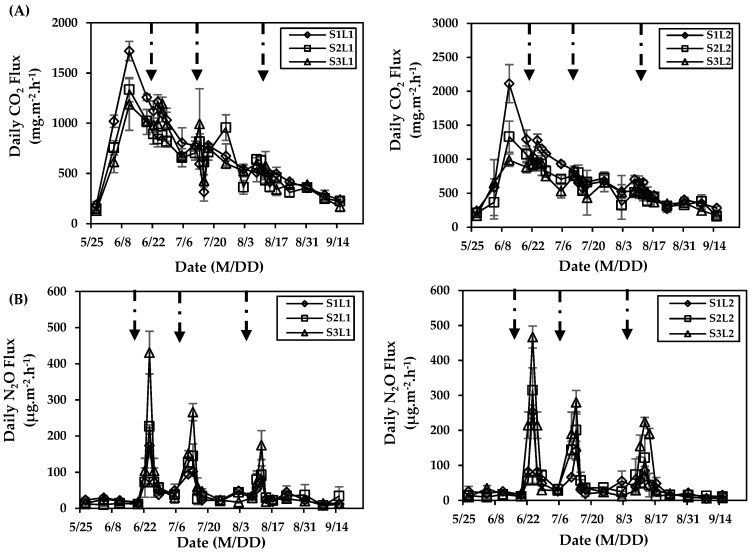
(**A**) Daily CO_2_ flux and (**B**) daily N_2_O flux during the growth period of maize for different irrigation water salinity (S1, S2, and S3 for 2 g/L, 3.5 g/L, and 5 g/L, respectively) and alternate irrigation regimes (L1 and L2 for one saline water cycle and two saline water cycles, respectively). Symbols represent the mean of three repeated tests and error bars represent standard deviation. Solid arrows indicate fertigation events.

Figure 5A shows the daily CO_2_ flux during the entire growth period. The trends of daily CO_2_ flux with time for all the treatments were similar. Due to previously high soil moisture, no irrigation was done before 29 May 2018. Therefore, the daily CO_2_ fluxes for all of the treatments before the first irrigation were similar. After the first irrigation on 29 May 2018, the daily CO_2_ flux for each treatment significantly increased, and the peak appeared after the third irrigation (9 June 2018) on 11 June 2018. In the L1 plots, the peak fluxes for S1, S2, and S3 were 1718.40 mg·m^−2^·h^−1^, 1333.94 mg·m^−2^·h^−1^, and 1186.45 mg·m^−2^·h^−1^, respectively. In the L2 plots, the peak fluxes for S1, S2, and S3 were 2112.41 mg·m^−2^·h^−1^, 1331.48 mg·m^−2^·h^−1^, and 986.53 mg·m^−2^·h^−1^, respectively. Subsequently, the daily CO_2_ flux for each treatment gradually decreased until the last measurement (15 September 2018).

Figure 5B represents the daily N_2_O flux during the growth period. The trends of daily N_2_O flux with time of all the treatments were similar. When there was no fertilization, the daily N_2_O flux was low. During the entire growth period, the peak N_2_O daily flux appeared after each of the three fertigations, and each appeared on the third day after fertilization. The larger N_2_O daily fluxes after each of the three fertigations were between 173.33 and 466.97 µg·m^−2^·h^−1^, 101.71 and 280.33 µg·m^−2^·h^−1^, and 78.67 and 224.22 µg·m^−2^·h^−1^, respectively. When compared to the L1 plots, the peak N_2_O daily fluxes in the L2 plots were larger. The differences after each fertigation ranged from 8.37–46.05% on 24 June 2018, 5.28–39.43% on 13 July 2018, and 8.06–32.13% on 12 August 2018.

Pearson correlation analyses between the mean gas emissions and the mean soil properties of the four main growth periods were conducted (Table 1). There was no significant correlation between the CO_2_ emission and the soil parameters (moisture, EC, and pH). Significant positive correlations were apparent between N_2_O emission and soil moisture, EC, and NO_3_^−^-N concentration, with a significant negative correlation between N_2_O emission and NH_4_^+^-N concentration.

### 3.3. Cumulative Greenhouse Gas Emission

Table 2 lists the cumulative greenhouse gas emissions in 2017 and 2018. In 2018, the cumulative CO_2_ emissions were greatest in S1 and least in S3 for both L1 and L2, but the cumulative N_2_O showed the opposite trend. The cumulative CO_2_ emissions in the L1 plots were greater than the L2 plots in general. The differences in S1, S2, and S3 were −3.02%, 5.83%, and 10.16%, respectively. The cumulative N_2_O emissions in the L2 plots were greater than the L1 plots. The differences in S1, S2, and S3 were 3.78 %, 1.69%, and 19.74%, respectively. The cumulative CO_2_ and N_2_O emissions increased in general when compared with 2017. As compared with 2017, the cumulative CO_2_ emissions increased by 22.33–44.73% and 29.58–56.85% in the L1 and L2 plots, respectively, and the cumulative N_2_O emissions increased by 2.95–47.85% and 5.27–95.13% in the L1 and L2 plots, respectively. Additionally, the cumulative N_2_O emissions decreased slightly in S1L1 as much as 1.56%.

Irrigation water salinity had a significant negative impact on cumulative CO_2_ emissions in both 2017 and 2018 (*p* < 0.05), but the alternate irrigation regime had no significant effect (*p* > 0.05). The significance analysis showed irrigation water salinity and alternate irrigation regime had no significant effect on cumulative N_2_O emissions in 2017 (*p* > 0.05), but irrigation water salinity had a significant effect in 2018 (*p* < 0.05).

### 3.4. Global Warming Potential

Table 3 shows the comprehensive global warming potential under all the treatments in 2017 and 2018. In 2018, the largest comprehensive GWP appeared in S1 in both the L1 and L2 plots. In the L1 plots, S1 was 13.28% and 15.04% greater than S2 and S3, respectively. In the L2 plots, S1 was 23.48% and 29.86% greater than S2 and S3, respectively. When compared with 2017, the comprehensive GWP of each treatment in 2018 increased as little as 21.93% and as much as 56.13%. The comprehensive GWP in S1 was highest among all irrigation water salinities in the same regime in both 2017 and 2018. In both years, the comprehensive GWP was greater in the L1 plots versus the L2 plots in general. The differences ranged from 2.74–18.36% in 2017, and ranged from −3.03–9.46% in 2018. The comprehensive GWP was only significantly affected by irrigation water salinity in both years (*p* < 0.05).

### 3.5. Maize Yield

Table 4 lists the maize yield under all treatments in 2017 and 2018. In both years, S1 showed the largest yield and S3 showed the least yield in both L1 and L2. In the L1 plots, S1 was 13.50% and 9.61% greater than S3 in 2017 and 2018, respectively. In the L2 plots, S1 was 11.71% and 18.55% greater than S3 in 2017 and 2018, respectively. When compared with 2017, maize yield increased in S1L1, S2L1, S3L1, and S1L2, and the largest increase appeared in S3L1 (5.27%). However, the maize yield decreased in S2L2 and S3L2, and the largest decrease appeared in S3L2 (8.45%). In both years, maize yield was greater in the L1 plots versus the L2 plots in general. The differences ranged from −3.10–5.49% in 2017, and ranged from 6.47–15.15% in 2018. Irrigation water salinity only significantly affected the maize yield in 2017 (*p* < 0.05), and by both irrigation water salinity and irrigation regime in 2018 (*p* < 0.05).

## 4. Discussion

The main processes that produced CO_2_ from soil are soil respiration with autotrophic (root) and heterotrophic (microbial) activity [11]. In the present study, CO_2_ emission from spring maize soil was mainly the product of soil respiration and maize respiration. The jointing stage and tasseling stage are the most vigorous stages of maize growth, with rapidly growing vegetative organs and strong respiration. The study found that the daily CO_2_ emissions of all treatments increased at the early growth of maize, reached the peak at 11 June 2018, and then decreased until physiological maturity stage, which is consistent with known trends of maize respiration. Some of the researchers found rapid rates of CO_2_ emission may be the result of heterotrophic consumption of relatively abundant labile carbon at the initial incubation period, and the slower rates appeared with the exhaustion of labile carbon in the final stages [27,28].

Many studies have found that soil moisture, EC, and pH were the most important regulating variables on soil CO_2_ emission rate [29,30,31]. However, in the present study, CO_2_ emission was not significantly correlated to any of the soil variables (soil moisture, EC, and pH). These results suggest that the soil sample was not large enough to reveal the correlation due to high spatial and temporal variability of CO_2_ emission from the spring maize soil.

The highest CO_2_ emission appeared in the plots treated with low salinity water in both 2017 and 2018, and the lowest at those that were treated with high salinity water under the same regime. For the alternate irrigation regime, two saline water cycles had lower CO_2_ emission than only one saline cycle, except for the 2 g/L irrigation water salinity in 2018. This suggests that CO_2_ emissions in the plots that were treated with low salinity water under different regimes are similar. In the present study, there was a negative correlation between irrigation water salinity and CO_2_ emission, which agrees with previous experiments [10,31]. The ion toxicity (Na^+^ specifically) [32], osmotic potential imbalance [30,33], or interaction of them [31] may reduce the activity of heterotrophic soil microorganisms, and then inhibit CO_2_ emission. However, some studies found the opposite to be true [34,35]. The varied types of salt in the soil may be the reason for the contradiction [36].

The production of N_2_O in the soil is a complex process, and nitrification and denitrification are the main sources of N_2_O in the soil, accounting for 63% of the total soil emissions [37]. Nitrification is an oxidation process under aerobic conditions, and denitrification is a reduction process under anaerobic conditions. Anthropogenic nitrogen input caused up to 3/4 of the annual total N_2_O in the plow land of China [38]. Fertilization directly brings nitrogen into the soil, which affects nitrification and denitrification and promotes N_2_O emissions. In this experiment, this observation was consistent with previous research [39]; N_2_O daily emission flux for each treatment significantly increased after each fertigation. The peak N_2_O daily emission after fertilization appeared on the third day, which is consistent with other researchers’ findings [14].

Changes in irrigation water salinity and alternate irrigation regime can lead to some changes in soil properties, such as soil moisture, EC, pH, and soil mineral N. The response of soil properties can change the environment of microbes and enzymes in the soil, which then influence N_2_O emission. O_2_ content will be less with higher soil moisture, inducing the production of N_2_O [40]. This may be the reason why there was a significant positive correlation between soil moisture and N_2_O emission in the present study. A possible explanation for the positive effect of EC on N_2_O emission is that, as the concentration of ions in the soil solution increases, the solubility of N_2_O decreases, and this is beneficial for N_2_O emission [41]. Soil pH represents the degree of acidity and alkalinity of the soil. Šimek, et al. [42] found that the value between 6.6 and 8.3 was the optimum pH for long-term denitrification, which explains the lack of significant correlation that was found here between soil pH and N_2_O emission. The correlation between soil NO_3_^−^-N and N_2_O emission is significantly positive, while a significantly negative correlation was found between soil NH_4_^+^-N and N_2_O emission. This suggests that nitrification had an important role in N_2_O emission. This result was consistent with [10], which found that nitrification could be responsible for N_2_O production in vegetation covered soil.

There are many studies about the impact of salinity on N_2_O emission. Some indicated that the correlation was positive [13,15], while some suggested that it was negative [10,43]; some found no correlation [44,45]. In this study, N_2_O emission was highest in the plots that were treated with high salinity water and lowest in those with low salinity water in each regime in 2018, while highest in the plots that were treated with low salinity water and the lowest in those with high salinity water in 2017. This shows the results were opposite in the first and second year. For the same irrigation water salinity, two saline water cycles had more N_2_O emissions from soil than only one saline cycle in 2018 and it was the opposite in 2017. There were significant differences between N_2_O emissions in 2018, while no significant difference in 2017. Only irrigation water salinity in 2018 had a significant effect on N_2_O emission. This suggests that irrigation water salinity has greater influence on N_2_O emission than an alternate irrigation regime, and salinity only affects N_2_O emissions when salt accumulation is high. Setschenow [46] suggested the solubility of N_2_O decreased as a result of increasing ionic strength of soil solution (Setschenow effect) and then N_2_O emissions increased. The present study showed that soil NH_4_^+^-N concentration was lower in soil that contained high levels of salt (Figure 4E), and soil NO_3_^−^-N concentration was higher in the soil with high levels of salt (Figure 4F). This indicates that high salinity promoted nitrification. However, high soil moisture in the soil containing high level salt (Figure 4A) led to the reduction of O_2_ content, thus, N_2_O production increased as a result of incomplete nitrification and cumulative N_2_O in soil under salt inhibition [15].

## 5. Conclusions

The Pearson correlations between soil properties and greenhouse gas emissions were studied. The analyses found that N_2_O emission was more sensitive to soil properties than CO_2_ emission. There was no significant correlation between CO_2_ emission and any soil properties. On the other hand, N_2_O emission had significant positive correlations with soil moisture, electrical conductivity, pH, and NO_3_^−^-N concentration, and significant negative correlation with NH_4_^+^-N concentration.

As the second year of conducting the field experiment, gas emissions of almost all treatments increased. Proportionally, using two saline water cycles had a larger increase. Year-to-year emissions increased with increasing irrigation water salinity. The smallest comprehensive global warming potential appeared at the 5 g/L salinity under two saline water cycles, which had the highest CO_2_ emission and the lowest N_2_O emission. Therefore, the treatment combining high irrigation water salinity and two saline water cycles has lesser ability to influence greenhouse gas emissions and climate change.

Maize yield should be another major factor to promote agricultural implication. It was found that 3.5 g/L salinity under one saline cycle, which showed smaller comprehensive global warming potential and higher maize yield, is the best irrigation choice.

The present study was conducted in an area of temperate continental climate, hoping to provide a foundation for applying different saline water under different alternate irrigation regimes. Similar studies should be conducted in other climate types to generalize the findings. In addition, it is also necessary to study the effects on greenhouse gas emissions under alternate irrigation regime while using different irrigation amounts, different fertilization amounts, and yield stability.

## Figures and Tables

**Table 1:** Pearson correlation analyses between the mean gas emissions and the mean soil properties of the four main growth periods.

	Moisture	EC	pH	**NH_4_^+^-N**	**NO_3_^−^-N**	CO_2_	N_2_O
Moisture	1	-	-	-	-	-	-
EC	0.448	1	-	-	-	-	-
pH	−0.128	−0.048	1	-	-	-	-
NH_4_^+^-N	−0.587 *	−0.473 *	0.019	1	-	-	-
NO_3_^−^-N	−0.110	0.317	0.399	−0.523 *	1	-	-
CO_2_	−0.121	−0.372	0.347	-	-	1	-
N_2_O	0.594 **	0.539 *	0.319	−0.758 **	0.503 *	-	1

* Significant at the 0.05 probability level. ** Significant at the 0.01 probability level. EC—conductivity.

**Table 2:** Cumulative greenhouse gas emission under different treatments *.

Treatments	Gases			
	CO_2_ (2017) (t·hm^−2^)	N_2_O (2017) (kg·hm ^2^)	CO_2_ (2018) (t·hm^−2^)	N_2_O (2018) (kg·hm ^2^)
S1L1	15,701.37 ± 1931.76 ^a^	1.02 ± 0.18 ^a^	19,206.77 ± 271.04 ^a^	1.01 ± 0.03 ^a^
S2L1	12,047.80 ± 985.34 ^b^	1.06 ± 0.06 ^a^	16,900.17 ± 363.35 ^b^	1.09 ± 0.08 ^bc^
S3L1	11,461.20 ± 1737.17 ^b^	0.87 ± 0.20 ^a^	16,587.64 ± 1204.97 ^bc^	1.28 ± 0.07 ^b^
S1L2	15,283.79 ± 1022.61 ^a^	0.99 ± 0.15 ^a^	19,805.29 ± 518.01 ^a^	1.04 ± 0.05 ^c^
S2L2	10,181.29 ± 828.66 ^b^	0.89 ± 0.09 ^a^	15,969.49 ± 1011.29 ^bc^	1.11 ± 0.14 ^bc^
S3L2	9840.09 ± 1003.86 ^b^	0.79 ± 0.06 ^a^	15,057.84 ± 708.78 ^c^	1.53 ± 0.09 ^a^

* Different lowercase letters (^a, b, c^) in the same column indicate that the difference reaches a significant level of 0.05 (ANOVA), and the same letters indicates the difference was not significant. The value is the mean value ± standard error. S1, S2, and S3 are for 2 g/L, 3.5 g/L, and 5 g/L irrigation water salinity, respectively. L1 and L2 are for one saline water cycle and two saline water cycles in the irrigation regime, respectively.

**Table 3:** Comprehensive global warming potential under different treatments [kg (CO_2_-C)·hm^−2^] *.

Year	Treatments					
	S1L1	S2L1	S3L1	S1L2	S2L2	S3L2
2017	15972.05 ± 1980.45 ^a^	12328.94 ± 1002.46 ^b^	11690.74 ± 1789.48 ^b^	15546.50 ± 1061.35 ^a^	10416.32 ± 851.45 ^b^	10048.33 ± 1018.94 ^b^
2018	19473.25 ± 280.15 ^a^	17189.61 ± 385.74 ^b^	16927.00 ± 1224.56 ^b^	20081.84 ± 530.17 ^a^	16263.83 ± 1049.12 ^b^	15464.18 ± 733.18 ^b^

* Different lowercase letters (^a, b^) in the same line indicate that the difference reaches a significant level of 0.05 (ANOVA), and the same indicate the difference was not insignificant. The value is the mean value ± standard error. S1, S2, and S3 are for 2 g/L, 3.5 g/L, and 5 g/L irrigation water salinity, respectively. L1 and L2 are for one saline water cycle and two saline water cycles in the irrigation regime, respectively.

**Table 4:** Maize yield under different treatments [kg·hm^−2^] *.

Year	Treatments					
	S1L1	S2L1	S3L1	S1L2	S2L2	S3L2
2017	15165.66 ± 660.52 ^a^	13676.09 ± 394.04 ^bc^	13361.77 ± 734.98 ^bc^	14376.26 ± 834.68 ^ab^	14113.59 ± 375.38 ^abc^	12869.31 ± 222.15 ^c^
2018	15418.40 ± 304.02 ^a^	14354.73 ± 891.39 ^ab^	14066.00 ± 595.84 ^b^	14481.80 ± 368.06 ^ab^	12920.57 ± 471.48 ^c^	12215.50 ± 163.97 ^c^

* Different lowercase letters (^a, b, c^) in the same line indicate that the difference reaches a significant level of 0.05 (ANOVA), and the same indicate the difference was not insignificant. The value is the mean value ± standard error. S1, S2, and S3 are for 2 g/L, 3.5 g/L, and 5 g/L irrigation water salinity, respectively. L1 and L2 are for one saline water cycle and two saline water cycles in the irrigation regime, respectively.
